# Ct-Perfusion Absolute Ghost Infarct Core Is a Rare Phenomenon Associated with Poor Collateral Status in Acute Ischemic Stroke Patients

**DOI:** 10.3390/jcm14092991

**Published:** 2025-04-25

**Authors:** Giorgio Busto, Andrea Morotti, Ilaria Casetta, Anna Poggesi, Davide Gadda, Andrea Ginestroni, Giorgio Arcara, Arianna Rustici, Andrea Zini, Alessandro Padovani, Enrico Fainardi

**Affiliations:** 1 Neuroradiology Unit, Department of Radiology, Careggi University Hospital, 50134 Florence, Italy; gaddad@aou-careggi.toscana.it (D.G.); a.ginestroni@gmail.com (A.G.); 2 Neurology Unit, Department of Clinical and Experimental Sciences, University of Brescia, 25121 Brescia, Italy; andrea.morotti85@gmail.com (A.M.); alessandro.padovani@unibs.it (A.P.); 3 IRCCS San Camillo Hospital, 30121 Venice, Italy; cti@unife.it (I.C.); giorgio.arcara@hsancamillo.it (G.A.); 4 Stroke Unit, Careggi University Hospital, 50134 Florence, Italy; anna.poggesi@unifi.it; 5 IRCCS Istituto delle Scienze Neurologiche di Bologna, UOSI di Neuroradiologia Ospedale Maggiore, 40100 Bologna, Italy; arianna.r87@gmail.com; 6 IRCCS Istituto delle Scienze Neurologiche di Bologna, UOC Neurologia e Rete Stroke Metropolitana, Ospedale Maggiore, 40100 Bologna, Italy; a.zini@ausl.bologna.it; 7 Neuroradiology Unit, Department of Experimental and Clinical Biomedical Sciences, University of Florence, 50134 Florence, Italy; enrico.fainardi@unifi.it

**Keywords:** acute ischemic stroke, absolute ghost infarct core, CT perfusion, collaterals, endovascular treatment, large vessel occlusion

## Abstract

**Background:** CT perfusion (CTP) overestimation of core volume >10 mL compared to the final infarct volume (FIV) size is the current definition of the ghost infarct core (GIC) phenomenon. However, subsequent infarct growth might influence FIV. We aimed to report a more reliable assessment of GIC occurrence, defined as no evidence of infarct at 24 h follow-up imaging, compared to CTP core volume at admission. This phenomenon was named absolute GIC (aGIC), and we investigated its prevalence and predictors. **Methods:** A total of 652 consecutive stroke patients with large vessel occlusion who achieved successful recanalization (mTICI 2b-3) after endovascular treatment (EVT) and non-contrast CT (NCCT) follow-up imaging at 24 h were retrospectively analyzed. Ischemic core volume was automatically generated from CTP, and FIV was manually determined on follow-up NCCT. Multivariable logistic regression was used to explore aGIC predictors. **Results:** We included 652 patients (53.3% female, median age 75 years), of whom 35 (5.3%) had an aGIC. The aGIC group showed higher ASPECTS (*p* < 0.001), shorter (<3 h) onset-to-imaging time (*p* < 0.016), poorer collaterals (*p* < 0.001), and higher hypoperfusion intensity ratio (*p* < 0.001) compared to the non-aGIC group. In multivariate analysis, ASPECTS (odds ratio (OR), 2.37; *p* <0.001), onset-to-imaging time (OR, 0.99; *p* = 0.034), collateral score (OR, 0.24; *p* < 0.001), and hypoperfusion intensity ratio (OR, 23.2; *p* < 0.001) were independently associated with aGIC. **Conclusions:** aGIC is a more reliable evaluation of infarct core volume overestimation assessed on admission CTP and represents a rare phenomenon, associated with ultra-early presentation and poor collaterals.

## 1. Introduction

Endovascular treatment (EVT) has dramatically changed the natural history of acute ischemic stroke (AIS) patients with large vessel occlusion (LVO), improving clinical outcome by the reperfusion of ischemic tissue at risk of infarction, namely penumbra, and limiting infarct core growth, that is, the irreversibly damaged neuronal tissue [1]. Different methods to define infarct core volume have been proposed based on two different EVT treatment windows [2,3]. Identifying the core size with perfusion imaging using relative cerebral blood flow (rCBF) is mandatory to assess eligible patients for EVT between 6 and 24 h from stroke onset [4]. Conversely, in patients up to 6 h from stroke onset, the use of advanced imaging is not recommended, to avoid delays due to the elaboration of perfusion maps from row data [5]. However, several studies have showed that patients who underwent EVT in the early time window selected with advanced imaging achieved better functional outcomes after successful recanalization than those who did not, encouraging the use of perfusion imaging in early presenters as well [6,7]. As a result, a precise calculation of core volume at presentation is pivotal to recruit more patients for EVT in the late time window and improve the clinical outcomes of patients treated in the early window when selected with perfusion imaging [7]. In this context, a possible overestimation of core extension by perfusion software has been described [8]. This condition, defined as ghost infarct core (GIC), has usually been considered as >10 mL of core size on admission CTP compared to the final infarct volume (FIV) visualized on non-contrast computed-tomography (NCCT) [9,10,11,12,13,14] or diffusion-weighted imaging (DWI) [13,15,16,17] at 24–48 h and up to 7 days [18] from stroke onset. In fact, median core overestimation was >10 mL also in two studies defining GIC as the difference between FIV and CTP-derived core volume ≤−1 mL [12,14]. Several mechanisms have been associated with the GIC phenomenon, but the presence of a short time from stroke onset to imaging [9,10,11,12,13,14,15,16,17,18] and poor collateral flow [12,16,17,18] are currently believed to be the most important. However, core overestimation differed across the studies in terms of incidence and impact on functional outcome [8,13], and it predominantly involved white matter [15,17]. On the other hand, FIV also demonstrated different impacts on clinical outcomes depending on the involvement of white or grey matter, irrespective of its size [19] In addition, although FIV is affected by brain edema formation in the early [20] and late [21] phases of infarct evolution, a significant number of AIS patients demonstrated a late infarct growth beyond 24 h even after successful EVT [22,23], suggesting that 24 h ischemic lesion volume measurement could not reliably estimate FIV [24,25]. Therefore, as patients with no early infarct growth commonly do not show late lesion expansion [24], a more reliable assessment of GIC could be obtained when a core volume visible on CTP is associated with the absence of infarcted tissue on 24 h follow-up imaging. In fact, only patients with infarcted tissue at admission and no infarct volume at 24 h certainly do not develop an infarct growth beyond 24 h and may be considered as true GIC subjects. We named this profile as absolute ghost infarct core (aGIC) and aimed to establish its frequency and determinants in a cohort of ischemic stroke patients. 

## 2. Methods

This cohort study was approved by the Ethical Committee of the University of Firenze (PN 26299). Written informed consent was obtained from each patient or from their legally authorized representatives at admission or waived by the institutional review board. STROBE (Strengthening the Reporting of Observational Studies in Epidemiology) guidelines for observational studies were utilized.

### 2.1. Patient Selection

We retrospectively analyzed a prospectively collected cohort of consecutive patients with AIS with anterior circulation LVO treated with EVT and admitted from January 2017 to September 2023 at Careggi University Hospital of Florence. All patients presenting with suspected AIS with LVO, and no history of renal failure or contrast allergy, routinely underwent NCCT, multi-phase CT-angiography (mCTA) of the cervical and intracranial vessels, and CTP at admission within 24 h of symptom onset. Patients were included if they presented at the emergency department with the following criteria: (1) NCCT Alberta Stroke Programme Early Computed Tomography Score (ASPECTS) ≥ 6; (2) diagnosis of AIS within 24 h from witnessed symptom onset or time last seen well; (3) evidence of internal carotid artery (ICA) and/or middle cerebral artery (MCA) M1 or M2 segment occlusion on CTA; (4) CTP performed at admission; (5) selected for receiving EVT; and (6) follow-up NCCT imaging performed at 24 ± 12 h. Exclusion criteria were: (1) NCCT ASPECTS < 6; (2) age < 18 years; (3) pregnancy; (4) severe pre-stroke disability defined as modified Rankin scale (mRS) ≥ 4; (5) detection of intracerebral hemorrhage (ICH) on admission NCCT; (6) contraindications to iodinated contrast agent; (7) poor quality of CT acquisition due to motion artifacts; and (8) inability to complete multi-modal CT protocol at baseline and/or 24 h follow-up NCCT.

### 2.2. Clinical Assessment

Clinical, demographic, and technical data were collected by trained investigators blinded to the outcomes of interest, from the patient’s medical records and a prospectively maintained institutional stroke database, including age, sex, pre-stroke functional status (mRS), the presence of stroke risk factors, the interval between symptom onset and neuroimaging, the initial National Institute of Health Stroke Scale (NIHSS) score, the use of intravenous thrombolysis (IVT), and EVT. Clinical outcome was measured using the mRS at 3 months. Good outcome was defined as mRS 0–2 at three months.

### 2.3. Imaging Acquisition

All imaging was conducted on 128-slice scanner (Philips Brilliance iCT, Best, The Netherlands). NCCT helical scans were performed from the skull base to the vertex using these following imaging parameters: 120 kV, 340 mA, 0.6 collimation, 1 s/rotation, table speed of 15 mm/rotation, 0.7 mL/kg contrast (maximum 90 mL), 5 to 10 s delay from injection to scanning, 120 kV, 251 mAs, 0.75 s/rotation, 0.8/0.4 mm thick slices (imbricated slices), and scan time 4 s. CTA covered from the carotid bifurcation to vertex. The second and third phase were acquired after a delay of 4 s that allows for table repositioning to the skull base. Scanning duration for each additional phase was 3.4 s. The axial images were reconstructed at 0.4 mm overlapping sections, and MIP multiplanar reconstructions for axial, coronal, and sagittal images of the circle of Willis were performed with 10 mm thickness at 3 mm intervals. CTP studies were obtained with a dynamic first-pass bolus-tracking methodology according to a two-phase imaging protocol, to avoid the truncation of time density curves, with toggling table technique. The two-phase acquisition consisted of a first phase every 3.2 s for 60 s and an additional second phase every 15 s for 113 s, which started 5 s after the automatic injection of 40 mL of non-ionic contrast agent followed by a saline flush of 40 mL at the rate of 4 mL/s. Sections of 8 cm length (across z axis) were acquired at 5 mm slice thickness. The other acquisition parameters were 80 kV, 150 mAs, and 0.33 rotation time. All CTP source images were reconstructed with the standard filter and display field of view (DFOV) of 25 cm.

### 2.4. Imaging Processing and Analysis

The extent of early ischemic changes was evaluated on baseline NCCT using the ASPECTS methodology [26]. mCTA collateral supply was graded by two diagnostic neuroradiologists, G.B. with more than 10 years of experience and E.F. with more than 20 years of experience, both blinded to clinical information and CTP outputs on a six-point scale according to a previously published scoring system [27]: grade 0 = zero filling in any phase in the affected territory; grade 1 = just a few vessels visible in any phase; grade 2 = delay of two phases and decreased prominence or number of vessels, or delay of one phase and some ischemic areas with no vessels; grade 3 = delay of two phases but the same prominence or number of vessels, or delay of one phase with the prominence or number of vessels significantly decreased; grade 4 = delay of one phase but prominence and extent are the same; grade 5 = no delay and normal or increased number or prominence of vessels. Grades 0–3 were considered as poor, and grades 4–5 as good collaterals [27]. M2 occlusions were considered LVO according to the current American Heart Association/American Stroke Association (AHA/ASA) guidelines [3]. Recanalization rate was assessed on digital subtraction angiography (DSA) at the end of endovascular treatment using the modified treatment in cerebral ischemia (mTICI) scale. Patients with mTICI score of 2b–3 were considered as successfully recanalized, whereas patients with mTICI scores ranging from 0 to 2a were classified as not [28]. CTP study was processed by commercially available delay-insensitive deconvolution automated software (Olea Sphere Version 3.0 SP23; Olea Medical, La Ciotat, France), using a standard singular value decomposition method according to manufacturer instructions. All steps, including motion correction, smoothing and evaluation of time density curves, and selection of arterial input and venous output functions, were checked for errors. As recommended by the vendor, total hypoperfused tissue and ischemic core volumes were defined as ischemic brain regions with Tmax threshold values >6 s (Tmax > 6 s) and relative cerebral blood flow threshold values less than 40% of normally perfused tissue (rCBF < 40%), respectively. The difference between Tmax > 6 s lesion extent and rCBF < 40% lesion size was considered as ischemic penumbra. Mismatch ratio was defined as the Tmax > 6 s volumes divided by the rCBF < 40% volume. Hypoperfusion intensity ratio (HIR) was defined as the ratio between Tmax > 10 s lesion and Tmax > 6 s lesion volumes, with HIR ≥ 0.4 predicting poor tissue-related collateral flow [29]. All these parameters were automatically segmented and calculated by the software. FIV was semi-automatically calculated with a multi-slice planimetric method using ITK-SNAP software (v.3.8.0-beta) on follow-up NCCT at 24 h after symptom onset/last known well blinded to the initial CTP data, EVT result, and clinical outcome. The presence of CTP infarct core at presentation in absence of FIV on 24 h NCCT follow-up imaging was considered as absolute GIC (aGIC).

### 2.5. Statistical Analysis

Categorical data were presented as absolute numbers (%), whereas continuous variables were summarized as mean ± standard deviation (SD) if normally distributed or median and interquartile ranges (IQR) in the case of non-parametric distribution. Baseline and treatment characteristics and clinical outcomes were compared between patients with and without aGIC using a two-tailed, independent-samples Student’s *t*-test or Mann–Whitney U-test as appropriate according to the data distribution for continuous variables. Dichotomous variables were compared using the chi squared test. aGIC was the outcome of interest, and its predictors were explored with logistic regression with backward elimination at *p*-values <0.1. Potential predictors were selected based on univariable analysis and the previous literature. The aGIC prediction model was based on clinical and imaging variables readily available on admission, in the hyperacute phase of stroke assessment. Furthermore, variables for which associations were seen in univariable analysis were included as adjustment factors as well. The analyses were performed considering aGIC as a dependent variable. Statistical analyses were performed using the software package IBM SPSS Statistics version 23. A *p*-value < 0.05 was considered statistically significant.

## 3. Results

**Figure 1:**
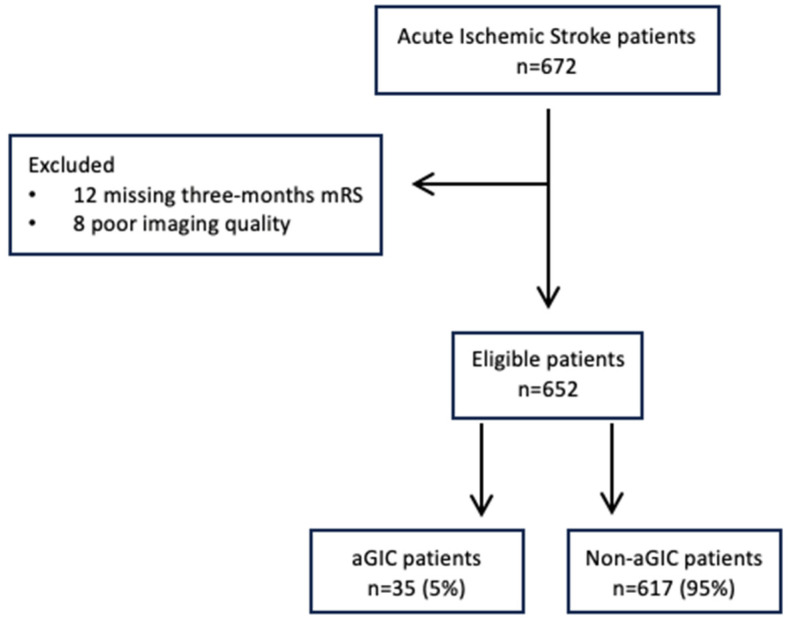
Flowchart of study population selection.

**Figure 2:**
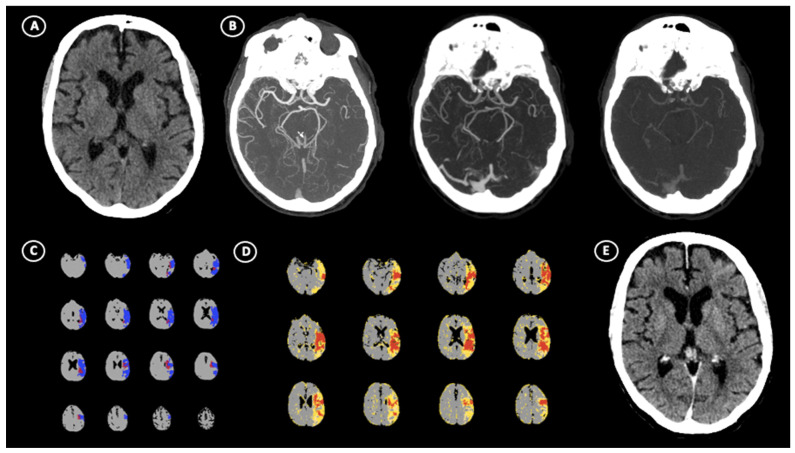
Illustrative case of absolute ghost infarct core (aGIC). A 64-year-old patient with acute ischemic stroke (AIS) suffering from the occlusion of left M1 segment of middle cerebral artery (MCA), which occurred within 3 h of stroke onset. (**A**) No visible hypodensity on non-contrast computed tomography (NCCT) resulted in ASPECTS = 10 with (**B**) poor multi-phase CT–angiography collaterals (Menon score = 2). CT perfusion shows the presence of 14.5 mL of core volume and 83.6 mL of penumbra volume in left hemisphere (**C**) and high hypoperfusion intensity ratio (HIR) = 0.7 (**D**). After successful recanalization, follow-up NCCT performed at 24 h (**E**) revealed no evidence of hypoattenuated areas, indicating the absence of final infarct volume.

A total of 652 patients undergoing the multimodal CT study protocol, successful EVT, and an NCCT at 24 h follow-up imaging were included in the analysis, as shown in Figure 1. The characteristics of the study population are summarized in Table 1. In total, 35 of 652 (5.3%) had aGIC. The median CTP aGIC volume was 26.2 mL (IQR 19.9–32.2). In the early time window, aGIC was seen in 32/394 (8.1%) patients. In the late time window, aGIC was found in 3/258 (1%) patients. In our cohort, full recanalization (mTICI 2b-3) was present in 535/652 (82%) patients, without any statistical differences between the aGIC and non-aGIC group. In the adjusted analysis (Table 2), variables that significantly differed between patients with and without aGIC were included in a binary logistic regression model with aGIC as a dependent variable. Patients with aGIC had better ASPECTS at admission (*p* < 0.001), shorter stroke onset-to-imaging time (*p* < 0.016), poorer collateral status (*p* < 0.001), higher hypoperfusion intensity ratio (*p* < 0.001), better NIHSS at discharge (*p* < 0.001), and better functional outcome (*p* = 0.023) than patients without aGIC. CTP Infarct core size and penumbra volumes at admission, onset-to-reperfusion time, and recanalization rate did not differ significantly between these two groups. Multivariable logistic regression analysis showed that ASPECTS (odds ratio (OR), 2.37; *p* < 0.001), onset-to-imaging time (OR, 0.99; *p* = 0.034), collateral score (OR, 0.24; *p* < 0.004), and hypoperfusion intensity ratio (OR, 23.2; *p* < 0.001) were independent predictors of aGIC after adjusting for potential confounders (Table 2). Figure 2 shows an illustrative case of aGIC.

## 4. Discussion

In this study, we defined ghost infarct core (GIC) as absolute GIC (aGIC) corresponding to the absence of final infarct lesion on 24 h NCCT follow-up imaging in the presence of CTP infarct core at presentation. Thus, aGIC proved to be an uncommon phenomenon, mainly affecting patients with ultra-early presentation within 3 h from symptom onset. Our approach could have relevant clinical implications as the adoption of advanced imaging in the early treatment window, up to 6 h from last known well, has been demonstrated to improve functional outcomes of subjects undergoing EVT [6,7]. Therefore, we provided evidence that aGIC occurrence did not significantly reduce CTP reliability as a selection tool for reperfusion therapies in AIS patients. In fact, as it is well known that infarct can expand over time for up to 5 days in both non-recanalized and recanalized subjects, [22,23] and ischemic volume assessed at 24 h may underestimate final infarct lesion in patients achieving full recanalization [24,25]. Although edema formation undoubtedly contributes to infarct growth in early [20] and, mainly, in delayed [21] phases of ischemic evolution, leading to an FIV overestimation, ischemic lesions increase after successful recanalization regardless of edema development [22]. Therefore, no infarct lesion at 24 h associated with infarct core visible on admission CTP, as indicated by aGIC, seems to be a more reliable method for establishing the presence of GIC, since no patients without infarcted tissue at 24 h developed delayed FIV [24]. This approach substantially differed from all other studies defining GIC as an increase of 10 mL from the infarct core volume calculated on admission CTP within 24–48 h [9,10,11,12,13,14,15,16,17] or up to 7 days [18]. As a consequence, in our study, we found that the incidence of absolute ghost infarct core in recanalized patients was about 5%, which was lower than in the majority of previous studies showing a frequency varying from 8.3% to 58.4% [9,10,11,12,13,14,15,16,18]. Of note, the frequency of GIC, defined as >10 mL of core size on admission CTP compared to the FIV calculated on 24 h NCCT follow-up, was 16.2% (106/652) in our population. In this regard, the most relevant difference was observed with studies adopting admission CBV maps to measure the infarct core size, in which ghost infarct core ranged from 38% to 58% of cases [9,11]. Taken together, these results suggest that CBV more commonly overestimates FIV than CBF maps, confirming the higher predictive ability of CBF in correctly identifying infarct core compared to CBV [30]. Conversely, although the definition of ghost infarct core was different, the prevalence of this condition obtained in ours and in a recent study [17] was similar. A possible explanation of these findings is that, in the investigation of Sarraj and associates, median FIV size at 24–48 h was 0.8 mL, very close to the zero value that we used for classifying ghost infarct core in the presence of an infarct lesion at admission CTP. Consistent with all previous studies [9,10,11,12,13,14,15,16,17,18], we found that ghost infarct core, as defined by aGIC, was significantly associated with a shorter time between symptom onset and the admission CTP, clearly prevailing in ultra-early presenters within 3 h from last known well. It is not surprising that aGIC was a time-dependent phenomenon since brain tissue tolerance to ischemic insult depends not only on the severity but also on the duration of ischemia [8,13,16], whereas decreased CBF values represent only the intensity but not the duration of hypoperfusion [8,13,17]. Therefore, as CTP is able to describe hemodynamic changes but not the viability of brain tissue [17,18], a short ischemia duration can allow neuronal cells to tolerate very low CBF levels without the development of cell death, leading to an infarct core overestimation by admission CTP [13]. In fact, lower CBF thresholds for better identifying infarct core were recently proposed in patients admitted early after symptom onset [17,31]. It is well known that the presence of poor collateral extent assessed with single-phase CTA (sCTA) and/or HIR is the main factor determining a larger infarct core at admission that is overestimated when onset-to-imaging time is short [12,16,17,18]. This overestimation of the infarct core in the ultra-early treatment window is mainly due to the greater tolerance that the brain tissue has towards ischemic damage compared to the degree of hypoperfusion that collaterals normally show [13]. Accordingly, we demonstrated that poor collaterals, as evaluated with mCTA and HIR, were significantly more represented in aGIC than in non-aGIC patients and were independently associated with aGIC. Compared to previous studies, our results appear more robust because they were obtained using both HIR for tissue-level and mCTA for pial arterial collateral measurements. In fact, while collaterals were graded with HIR in some publications [12,18], in other investigations, they were scored with sCTA [16] or with HIR and sCTA [17]. However, it is currently accepted that mCTA is the more reliable tool for establishing pial arterial collateral filling [32]. Collateral assessment with mCTA was used only in the study of Ospel and coworkers [13], in which ghost infarct core was associated with good and not with poor collaterals, probably because, unlike Menon score [28], moderate collaterals were considered as good. Finally, in line with three prior publications [12,13,14,16], in our study, aGIC was associated with good functional outcome. This was an expected finding due to no evidence of FIV at 24 h from onset characterizing our population. Nevertheless, the relationship between ghost infarct core and FIV was not reported in another study [18], confirming that FIV does not necessarily correlate to clinical outcome [33]. Our study has some limitations. First, this was a retrospective, single-center analysis requiring a prospective validation. Second, the non-randomized design of our study introduced potential bias by unmeasured confounders. Third, generalizability might be limited as our analysis was restricted to patients selected for EVT. Fourth, we did not perform a further evaluation of subsequent follow-up imaging beyond NCCT at 24 h. Finally, as recommended by the vendor, admission infarct core volume was calculated using rCBF < 40% as the threshold value instead of rCBF < 30%, which is widely considered the reference threshold value. Therefore, a confirmation of our findings using other platforms is needed. In addition, we did not perform a subgroup analysis with rCBF <20% to verify whether the incidence of ghost infarct core was limited by the use of lower CBF thresholds as previously suggested [17,18], since the corresponding CBF thresholds for Olea software have not yet been identified.

## 5. Conclusions

Ghost infarct core, as indicated by aGIC, is an uncommon phenomenon in ultra-early presenters associated with poor collaterals imaged within 3 h after onset. The use of an absolute definition of GIC might provide a more reliable assessment of infarct core volume overestimation observed on admission CTP. 

## Figures and Tables

**Table 1:** Clinical and neuroimaging characteristics of patient population.

Variable	Patients With Absolute Ghost Core	Patients Without Absolute Ghost Core
	n = 35	n = 617
Age, mean (±SD), years	76.0 (12.3)	74.5 (12.9)
Sex, woman, n (%)	18 (51.4)	330 (53.4)
Admission NIHSS, median (IQR)	16 (9–21)	18 (12–22)
ASPECTS, median (IQR)	10 (9–10)	8 (7–9)
tPA before EVT, n (%)	16 (45.7)	248 (40.1)
Time from onset to NCCT, minutes, median (IQR)	160 (90–250)	290 (192–555)
Time from onset to NCCT <6 h, n (%)	32 (91.4)	362 (58.6)
Time from onset to NCCT >6 h, n (%)	3 (8.6)	255 (41.4)
Occlusion site, left n (%)	23 (65.7)	333 (53.9)
M1 segment, n (%)	19 (54.3)	364 (58.9)
M2 segment, n (%)	9 (25.7)	113 (18.4)
ICA segment, n (%)	7 (20.0)	140 (22.7)
Collateral score, median (IQR)	3 (3–4)	4 (3–4)
Poor, n (%)	23 (71.4)	227 (36.7)
Good, n (%)	12 (28.6)	390 (63.3)
Hypoperfusion intensity ratio, median (IQR)	0.50 (0.30–0.60)	0.34 (0.20–0.50)
Infarct core (rCBF<40%) at baseline in mL, median (IQR)	26.2 (19.9–32.2)	23.7 (9.9–44.3)
Ischemic penumbra volume, median (IQR), mL	65.0 (39.0–109.3)	56.4 (28.5–93.1)
mTICI score 2b–3, n (%)	35 (100.0)	500 (81.0)
Onset to reperfusion time, minutes, median (IQR)	330 (265–385)	425 (310–715)
Infarct volume at 24 h in mL, median (IQR)	0 (0–0)	29.5 (15.3–69.6)
NIHSS at 24 h, median (IQR)	4 (2-8)	13 (5-20)
mRS at three-months 0-2, n (%)	23 (65.7)	270 (43.7)

SD indicates standard deviation; NIHSS, National Institutes Health Stroke Scale; IQR, interquartile range; ASPECTS, Alberta Stroke Program Early CT Score; tPA, tissue plasminogen activator; EVT, endovascular treatment; NCCT, non-contrast computed tomography; ICA, internal carotid artery; rCBF, relative cerebral blood flow; mTICI, modified thrombolysis in cerebral infarction; mRS, modified Rankin scale.

**Table 2:** Predictors of absolute ghost infarct core.

	Univariable		Multivariable	
	OR (95% CI)	*p*	OR (95% CI)	*p*
Age, years	1.02 (0.98–1.07)	0.222		
Sex, woman	0.82 (0.29–2.33)	0.714		
Admission NIHSS	1.05 (0.97–1.14)	0.192		
ASPECTS	2.36 (1.52–3.67))	<0.001	2.37 (1.61–3.48)	<0.001
tPA before EVT	0.67 (0.25–1.77)	0.426		
Onset-to-CT time	0.99 (0.98–0.99)	0.016	0.99 (0.99–1.01)	.034
Onset-to-reperfusion time	1.00 (0.99–1.01)	0.116		
mTICI score 2b-3	0.86 (0.31–2.56)	0.996		
Collateral score	0.21 (0.10–0.42)	<0.001	0.24 (0.12–0.45)	<0.001
Hypoperfusion intensity ratio	8.88 (2.9–27.0)	<0.001	23.2 (1.54–48.9)	<0.001
Infarct core (rCBF <40%)	0.97 (0.95–1.00)	0.100		
Ischemic penumbra volume	0.99 (098–1.01)	0.365		
NIHSS at 24 h	0.80 (0.72–0.89)	<0.001		
mRS at three months 0–2	1.11 (0.38–3.25)	0.023		

OR indicates odds ratio; CI, confidence interval; NIHSS, National Institutes Health Stroke Scale; ASPECTS, Alberta Stroke Program Early CT Score; tPA, tissue plasminogen activator; EVT, endovascular treatment; mTICI, modified thrombolysis in cerebral infarction; rCBF, relative cerebra blood flow; mRS modified Rankin scale.

## Data Availability

The data that support the findings of this study are available upon reasonable request.
